# Comparison of Short-Wavelength Reduced-Illuminance and Conventional Autofluorescence Imaging in Stargardt Macular Dystrophy

**DOI:** 10.1016/j.ajo.2016.06.003

**Published:** 2016-08

**Authors:** Rupert W. Strauss, Beatriz Muñoz, Anamika Jha, Alexander Ho, Artur V. Cideciyan, Melissa L. Kasilian, Yulia Wolfson, SriniVas Sadda, Sheila West, Hendrik P.N. Scholl, Michel Michaelides

**Affiliations:** a Wilmer Eye Institute, Johns Hopkins University, Baltimore, Maryland; b Moorfields Eye Hospital, London, United Kingdom; c University College London, Institute of Ophthalmology, London, United Kingdom; d Doheny Image Reading Center, Los Angeles, California; e Scheie Eye Institute, Perelman School of Medicine at the University of Pennsylvania, Philadelphia, Pennsylvania; f David Geffen School of Medicine at University of California Los Angeles, Los Angeles, California

## Abstract

**Purpose:**

To compare grading results between short-wavelength reduced-illuminance and conventional autofluorescence imaging in Stargardt macular dystrophy.

**Design:**

Reliability study.

**Methods:**

setting: Moorfields Eye Hospital, London (United Kingdom). patients: Eighteen patients (18 eyes) with Stargardt macular dystrophy. observation procedures: A series of 3 fundus autofluorescence images using 3 different acquisition parameters on a custom-patched device were obtained: (1) 25% laser power and total sensitivity 87; (2) 25% laser power and freely adjusted sensitivity; and (3) 100% laser power and freely adjusted total sensitivity (conventional). The total area of 2 hypoautofluorescent lesion types (definitely decreased autofluorescence and poorly demarcated questionably decreased autofluorescence) was measured. main outcome measures: Agreement in grading between the 3 imaging methods was assessed by kappa coefficients (κ) and intraclass correlation coefficients.

**Results:**

The mean ± standard deviation area for images acquired with 25% laser power and freely adjusted total sensitivity was 2.04 ± 1.87 mm^2^ for definitely decreased autofluorescence (n = 15) and 1.86 ± 2.14 mm^2^ for poorly demarcated questionably decreased autofluorescence (n = 12). The intraclass correlation coefficient (95% confidence interval) was 0.964 (0.929, 0.999) for definitely decreased autofluorescence and 0.268 (0.000, 0.730) for poorly demarcated questionably decreased autofluorescence.

**Conclusions:**

Short-wavelength reduced-illuminance and conventional fundus autofluorescence imaging showed good concordance in assessing areas of definitely decreased autofluorescence. However, there was significantly higher variability between imaging modalities for assessing areas of poorly demarcated questionably decreased autofluorescence.

Stargardt macular dystrophy is the most common form of juvenile macular degeneration.^1^, ^2^ It is an autosomal recessively inherited disorder caused by disease-causing variants in the *ABCA4* gene, encoding a photoreceptor-specific ATP-binding cassette transporter involved in active transport of all-*trans*-retinal across the disc membranes within photoreceptor outer segments.^3^ Failure of this process leads to the accumulation of N-retinylidene-N-retinyethanolamine (A2E), one of the major components of lipofuscin in the retinal pigment epithelium.^4^ High concentrations of N-retinylidene-N-retinyethanolamine and lipofuscin are believed to be cytotoxic, leading to dysfunction and cell death of the retinal pigment epithelium and photoreceptors. Clinically, one of the early hallmarks of Stargardt macular dystrophy is retinal “flecks,” which represent areas of lipofuscin accumulation. Flecks can resorb over time, and with disease progression, macular atrophy occurs with deterioration of retinal function.^5^ These characteristic features of Stargardt macular dystrophy can be easily and noninvasively imaged by confocal scanning laser fundus autofluorescence using signals originating from fluorophores (such as lipofuscin) within the retina and the retinal pigment epithelium after excitation by short-wavelength light.^6^ The accumulation of lipofuscin leads to areas of increased autofluorescence, whereas areas of atrophy are associated with decreased autofluorescence.^5^ Since its early descriptions,^7^, ^8^ fundus autofluorescence has been widely explored, and recent technological advances, especially the introduction of confocal laser ophthalmoscopy and frame averaging techniques, have led to a significantly improved signal-to-noise ratio and enhanced quality of fundus autofluorescence images.^6^

For these reasons, and because the U.S. Food and Drug Administration is considering fundus autofluorescence as a possible surrogate endpoint for clinical trials,^9^ fundus autofluorescence has been chosen as the primary outcome measure in the “Natural History of the Progression of Stargardt Disease: Retrospective and Prospective Studies” (ProgStar; ClinicalTrials.gov Identifier NCT01977846). These multicenter studies aim to characterize the natural course of Stargardt macular dystrophy and to validate possible outcome measures for emerging clinical trials including gene therapy, stem cell therapy, and pharmacotherapy.^10^

However, the appropriate fundus autofluorescence imaging protocols remain controversial, in terms of both limiting potential toxicity from short-wavelength light and ensuring optimum image quality and thereby measurement sensitivity to monitor disease progression. One mechanism of the aforementioned cytotoxicity of N-retinylidene-N-retinyethanolamine is its mediation through blue light–induced damage to retinal pigment epithelial cells by photooxidative damage.^11^, ^12^ The high-intensity and short-wavelength excitation light used in conventional fundus autofluorescence imaging could, at least in principle, increase the rate of lipofuscin accumulation and/or its toxicity.^13^ Cideciyan and associates were the first to describe the concept of using short-wavelength reduced-illuminance autofluorescence imaging in *ABCA4*-associated retinopathy to reduce potential toxicity^13^; however, the question remains whether image integrity is compromised. Although short-wavelength reduced-illuminance autofluorescence imaging results have been reported to be both qualitatively and quantitatively comparable to those in the literature on conventional fundus autofluorescence imaging, one described practical shortcoming was noisier and darker images apparent on the acquisition screen, leading to more difficult imaging.^13^

In the context of the ProgStar study, we acquired images where the power of the imaging laser beam was reduced to 25% of its conventional setting. For this purpose, a special software tool was developed and provided by Heidelberg Engineering (Heidelberg, Germany) to all participating sites in the ProgStar study.^10^

The purpose of this study is therefore to compare the grading results of images obtained using the short-wavelength reduced-illuminance autofluorescence imaging method with those obtained with conventional fundus autofluorescence imaging in the same patient cohort and determine the correlation and areas of disagreement. This has implications for both routine clinical care and clinical trial endpoint design.

## Methods

This reliability study was approved by the local Ethics Committee of Moorfields Eye Hospital, NHS Foundation Trust; adhered to the provisions of the Declaration of Helsinki; and complied with the Health Insurance Portability and Accountability Act. Ethics committee approval for the ProgStar study was granted by the Western Institutional Review Board, the local Ethics Committee of Moorfields Eye Hospital, NHS Foundation Trust, the institutional review board of Johns Hopkins University School of Medicine, and the Human Research Protection Office of the U.S. Army Medical Research & Materiel Command, prior to enrollment of the first patient, respectively. These studies have been registered at www.clinicaltrials.gov (Identifier NCT01977846). Written informed consent was obtained by all participants and (if applicable) their guardians prior to enrollment.

### Subjects

Inclusion criteria were as follows:

• Age of at least 6 years
• Molecularly confirmed Stargardt macular dystrophy with at least 2 likely disease-causing mutations in *ABCA4*; if only 1 mutation was present, the patient had to have the typical phenotype for Stargardt macular dystrophy (ie, flecks at the level of the retinal pigment epithelium)
• Atrophic lesion of at least 300 μm in diameter; all lesions together must total less than 5 standard disc diameters (= 12.00 mm^2^)
• Clear ocular media and adequate pupillary dilation to permit good-quality fundus autofluorescence imaging
• Participation in the prospective ProgStar study.

Exclusion criteria were as follows:

• Other ocular disease, such as choroidal neovascularization, diabetic retinopathy, and degenerative retinal dystrophies other than Stargardt macular dystrophy
• Intraocular surgery 90 days prior to the imaging visit
• Current or previous participation in an interventional study for Stargardt macular dystrophy, such as gene or stem cell therapy.

### Study Design and Image Acquisition

Pupils were dilated with 1% tropicamide and 2.5% phenylephrine hydrochloride and fundus autofluorescence images were acquired using a Spectralis FA + OCT device (Heidelberg Engineering, Heidelberg, Germany). The device was equipped with a custom-developed software tool provided by Heidelberg Engineering to reduce laser intensity during acquisition of fundus autofluorescence images. Using this software tool, the laser power can be reduced to 25%, 50%, or 75% of the preset 100% laser power. Three images with different acquisition parameters were obtained in the study eye of each patient by 1 of 4 experienced photographers certified for the ProgStar study. All images were acquired with a 30-degree field of view in the high-speed mode centered on the anatomic fovea, not normalized, and an automatic real-time (ART) averaging of ≥15 frames. Differences in the parameter settings were in the laser power and total sensitivity: (1) the first image was obtained with a laser power of 25% and a fixed total sensitivity of 87 over an imaging duration of ∼5 seconds; (2) the second image was obtained with 25% laser power; however, total sensitivity was not fixed, but adjusted by the photographer to optimize image illumination (“freely adjusted”); (3) the last image was obtained with laser power 100% (conventional) and total sensitivity that was adjusted for an optimal image exposure (“freely adjusted”) over an imaging duration of ∼30 seconds. The 25% setting corresponds to a retinal illuminance of 2.5 × 10^5^ scot-trolands and the 100% setting to 10 × 10^5^ scot-trolands. These 2 settings would be predicted to result in rhodopsin bleaches of 12% and 95%, respectively, in normal eyes. Time interval between each capture of images was at least 5 minutes.

### Image Grading and Analysis

Deidentified images were sent to the Doheny Imaging Reading Center, David Geffen School of Medicine at the University of California Los Angeles, Los Angeles, California, and graded using a semi-automated software tool (Heidelberg Engineering RegionFinder). The quality of the images submitted for grading was assessed by evaluating focus and clarity (“blurriness” or “fuzziness”).

#### Quantitative Parameters

Three different categories of areas of decreased autofluorescence were graded.^14^ The reference points for a scale of decreased autofluorescence were the optic nerve head and blood vessels as “100% level of darkness” and the peripheral retinal background fundus autofluorescence as “0% level of darkness.” The term “definitely decreased autofluorescence” was defined for areas in which the level of darkness was close to 100% (at least 90%) in reference to the optic nerve head/blood vessels; regions with levels between 50% and 90% darkness were defined as “questionably decreased autofluorescence”; in these lesions, the sharpness of the corresponding lesion border defined a lesion either as “well-demarcated questionably decreased autofluorescence” or “poorly demarcated questionably decreased autofluorescence.” In cases with multiple types of lesion, the areas of all subtypes were summed, respectively. Images were independently graded by 2 reading center–certified graders, with at least 1 grader being a senior-level grader. Discordant initial assessments underwent adjudication by a reading center investigator if consensus could not be reached.

#### Qualitative Parameters

Qualitative parameters included the following: (1) focus and clarity of images; (2) presence of increased autofluorescence at the lesion edge; (3) background uniformity (a homogeneous background was defined as an even distribution of background autofluorescence; a heterogeneous background signal was defined as widespread small foci of increased or reduced autofluorescence, as previously described^5^); and (4) presence of flecks.

### Statistical Analysis

The primary comparisons were areas of decreased autofluorescence (definitely decreased autofluorescence, well-demarcated questionably decreased autofluorescence, and poorly demarcated questionably decreased autofluorescence) in the 3 different image acquisition settings. Kappa coefficients (κ) were used for the assessment of intergrader agreement in qualitative grading (eg, the presence/absence of each type of decreased autofluorescence, background uniformity, etc) in the respective image, and kappa coefficients >0.61 were considered to be indicative of good agreement. Intraclass correlation coefficients (ICC) with 95% confidence intervals (CI) were used for the calculation of quantitative assessments (comparison between different image acquisition parameters). Differences in area sizes were compared using the paired *t* test. Statistical analyses were performed in SAS Statistical Analysis Software Version 9.4 (SAS Institute, Cary, North Carolina, USA) and R version 2.15.1 (The R Foundation for Statistical Computing, Vienna, Austria).

## Results

**Figure 1:**
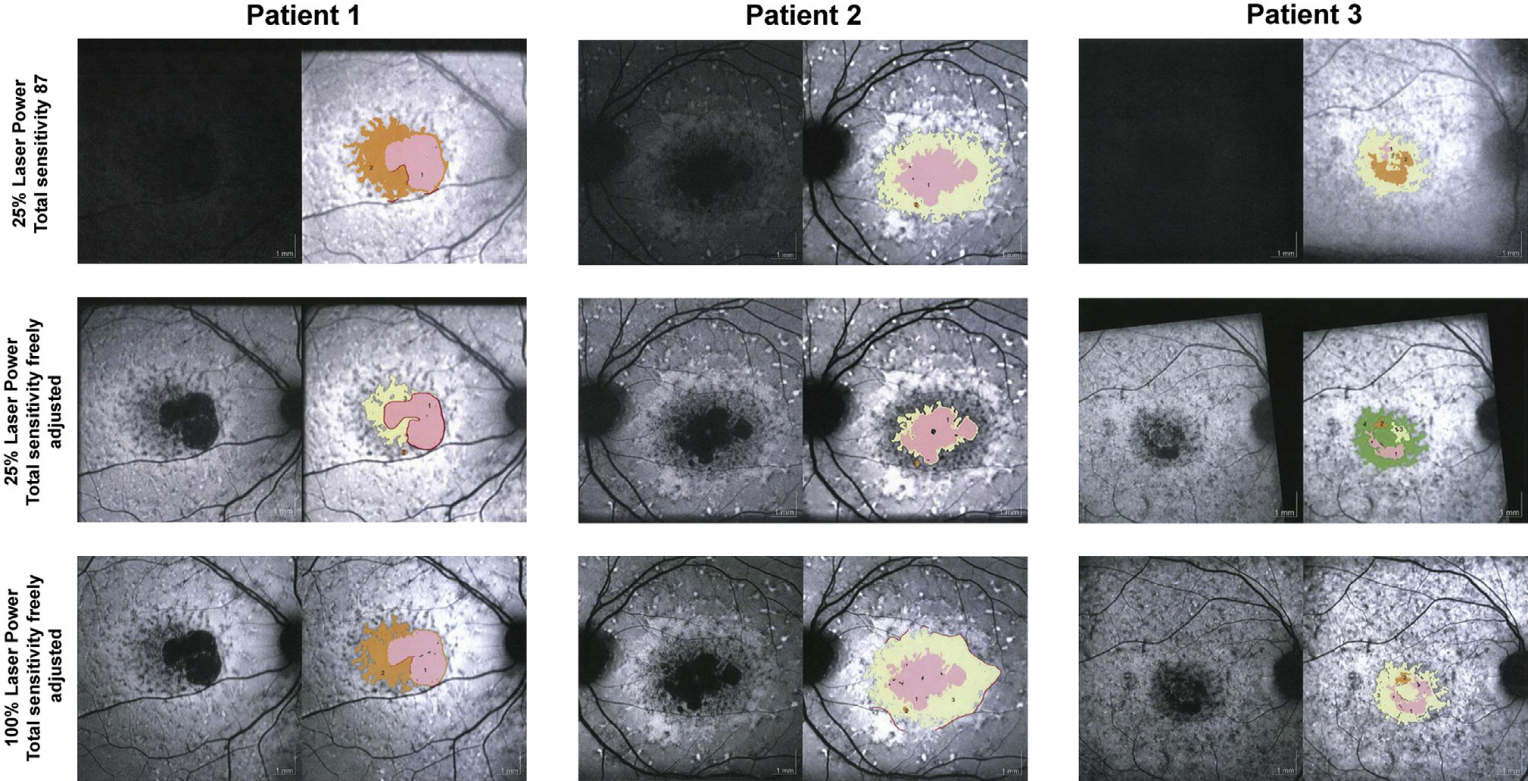
Shown are 3 eyes of 3 representative patients (Left column, Patient 1; Middle column, Patient 2; Right column, Patient 3) imaged with 3 different parameter settings (Top row: 25% laser power, total sensitivity 87; Middle row: 25% laser power, freely adjusted total sensitivity; Bottom row: 100% laser power, freely adjusted total sensitivity) and the respective grading of definitely decreased autofluorescence and poorly demarcated questionably decreased autofluorescence as provided by the Heidelberg RegionFinder tool. These images were contrast- and shadow-corrected by the software. Patient 1: (Top left) Total area of definitely decreased autofluorescence (pink) was 4.24 mm^2^, total area of poorly demarcated questionably decreased autofluorescence (orange) was 5.22 mm^2^. (Middle left) Total area of definitely decreased autofluorescence (pink) was 3.92 mm^2^, total area of poorly demarcated questionably decreased autofluorescence (yellow) was 2.55 mm^2^. (Bottom left) Total area of definitely decreased autofluorescence (pink) was 4.20 mm^2^, total area of poorly demarcated questionably decreased autofluorescence (orange) was 3.91 mm^2^. Patient 2: (Top middle) Total area of definitely decreased autofluorescence (pink and orange) was 4.42 mm^2^, total area of poorly demarcated questionably decreased autofluorescence (yellow) was 8.03 mm^2^. (Center) Total area of definitely decreased autofluorescence (pink and orange) was 3.26 mm^2^, total area of poorly demarcated questionably decreased autofluorescence (yellow) was 1.35 mm^2^. (Bottom middle) Total area of definitely decreased autofluorescence (pink and orange) was 4.15 mm^2^, total area of poorly demarcated questionably decreased autofluorescence (yellow) was 8.97 mm^2^. Patient 3: (Top right) Total area of decreased autofluorescence (pink and orange) was 1.39 mm^2^, total area of poorly demarcated questionably decreased autofluorescence (yellow) was 3.60 mm^2^. (Middle right) Total area of definitely decreased autofluorescence (pink, orange, and yellow) was 1.11 mm^2^, total area of poorly demarcated questionably decreased autofluorescence (green) was 3.31 mm^2^. (Bottom right) Total area of definitely decreased autofluorescence (pink and orange) was 1.24 mm^2^, total area of poorly demarcated questionably decreased autofluorescence (yellow) was 3.33 mm^2^.

Eighteen eyes of 18 patients were enrolled in this reliability study at Moorfields Eye Hospital, London (United Kingdom). If both eyes of a patient were eligible and enrolled into the ProgStar study, 1 eye was randomly chosen for the purpose of this study. Mean age (± standard deviation) was 38.3 (± 14.2) years (Supplemental Table 1; Supplemental Material available at AJO.com). Figure 1 provides illustrative examples of images obtained with all 3 different parameter settings and respective grading results of areas of definitely decreased autofluorescence and poorly demarcated questionably decreased autofluorescence.

### Presence/Absence of Categories of Decreased Autofluorescence

Based on the grading of images acquired with 25% laser power and 87 total sensitivity (short-wavelength reduced-illuminance autofluorescence imaging), 15 eyes had definitely decreased autofluorescence lesions and 12 eyes had poorly demarcated questionably decreased autofluorescence lesions. Only 1 eye of 1 patient showed a lesion of well-demarcated questionably decreased autofluorescence (Table 1). Based on the grading of images acquired with 25% laser power and *freely adjusted* total sensitivity (short-wavelength reduced-illuminance autofluorescence imaging), 15 eyes had definitely decreased autofluorescence lesions, 12 eyes had poorly demarcated questionably decreased autofluorescence lesions, and no eyes had lesion(s) of well-demarcated questionably decreased autofluorescence (Tables 1 and 2). In contrast, with conventional fundus autofluorescence, 14 eyes had definitely decreased autofluorescence lesions and 12 eyes had poorly demarcated questionably decreased autofluorescence lesions. One eye had lesion(s) of well-demarcated questionably decreased autofluorescence (Tables 1 and 2).

### Area of Definitely Decreased Autofluorescence

**Figure 2:**
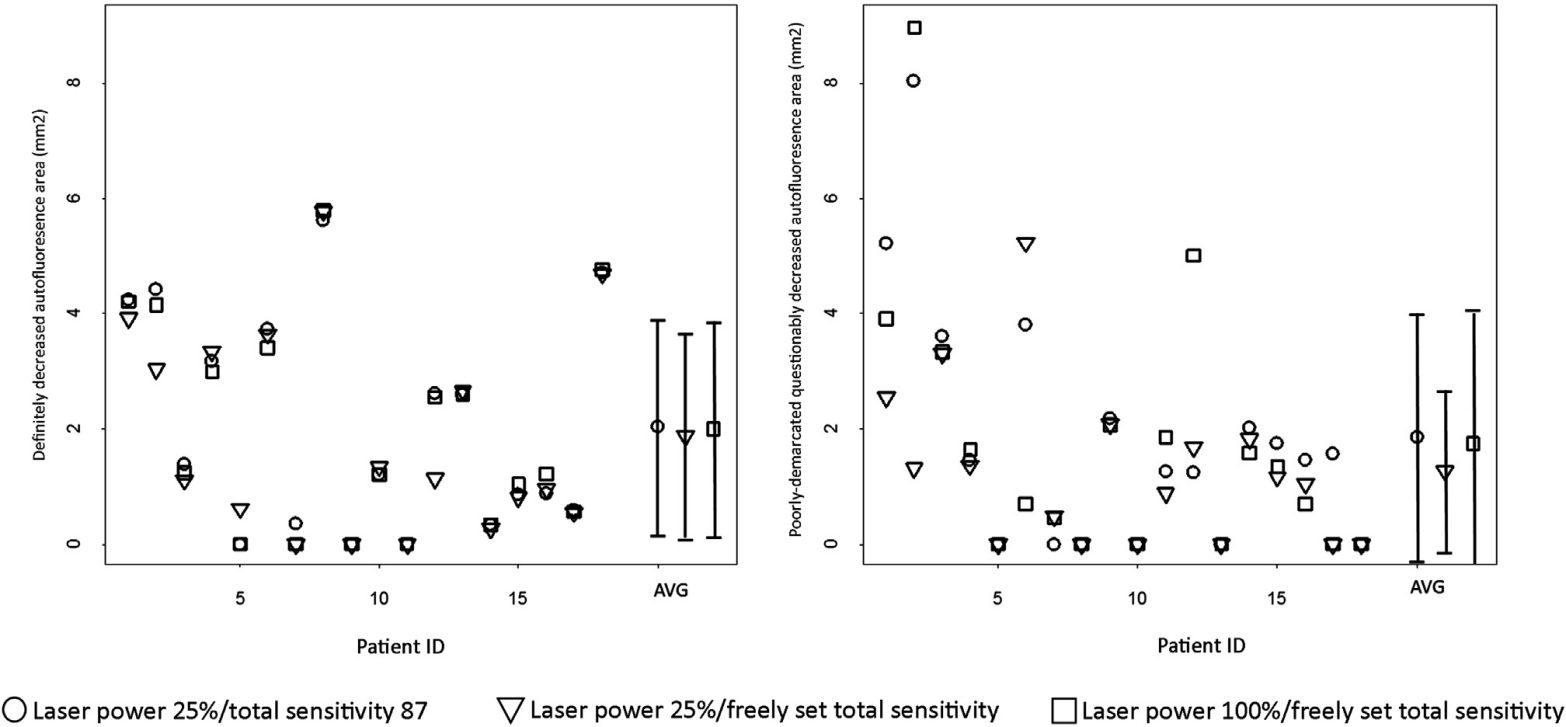
Measurements of areas of definitely decreased autofluorescence (Left) and poorly demarcated questionably decreased autofluorescence (Right) in the 18 eyes of 18 participants enrolled into this study. Images were acquired with 3 different parameter settings: 2 images were obtained with short-wavelength reduced-illuminance autofluorescence imaging using 25% laser power/total sensitivity 87 and 25% laser power/freely adjusted total sensitivity, and 1 image was obtained with conventional fundus autofluorescence imaging (100% laser power and freely adjusted total sensitivity). AVG = average and standard deviation (bars) of area measurements; areas of absent decreased autofluorescence (zeros) were included, respectively.

The mean ± standard deviation (SD) for the area of definitely decreased autofluorescence was 2.04 ± 1.87 mm^2^ in images obtained with 25% laser power/total sensitivity 87, 1.88 ± 1.79 mm^2^ in images obtained with 25% laser power/freely adjusted total sensitivity, and 2.00 ± 1.86 mm^2^ in images obtained with conventional fundus autofluorescence. Figure 2 (Left) illustrates the measurements of areas of definitely decreased autofluorescence using the 3 different acquisition settings. These measurements of definitely decreased autofluorescence derived from all 3 acquisition settings were highly correlated with each other (Table 3); the ICC (95% CI) was 0.964 (0.929, 0.999) when comparing images acquired at 25% laser power/freely adjusted total sensitivity and conventional fundus autofluorescence. There was no statistically significant difference in determination of area size between the 3 acquisition parameter settings (Table 3); moreover, when comparing image acquisition settings with freely adjusted total sensitivity (laser power 25% and laser power 100%), the mean ± SD difference in area between the 2 modalities was −0.124 (0.473) mm^2^ (*P* = .28). In a previous study for measuring areas of definitely decreased autofluorescence, ICC for intergrader agreement was 0.993 (0.986, 0.996) using the semi-automated software tool (Heidelberg RegionFinder) and the ICC for intragrader agreement was 0.981 (0.963, 0.990) using the same semi-automated software tool (Heidelberg RegionFinder).^14^

### Area of Poorly Demarcated Questionably Decreased Autofluorescence

Figure 2 (Right) illustrates the measurements of areas of poorly demarcated questionably decreased autofluorescence using the 3 different acquisition settings. The mean ± standard deviation for areas of poorly demarcated questionably decreased autofluorescence as determined with the 3 different acquisition parameters was more variable (n = 12 eyes). The greatest area was 1.86 ± 2.14 mm^2^ in images obtained with 25% laser power/total sensitivity 87. The lowest was 1.27 ± 1.40 mm^2^ in images obtained with 25% laser power/freely adjusted total sensitivity. In images obtained with conventional fundus autofluorescence, the area of poorly demarcated questionably decreased autofluorescence was 1.75 ± 2.33 mm^2^; the differences were not statistically significant (Table 4).

There was variation in the ICCs of measured lesion areas between these different image acquisition parameters and also for the detected differences in area sizes (Table 4). The ICC was 0.268 (0.000, 0.730) for poorly demarcated questionably decreased autofluorescence for the comparison between images obtained at 25% laser power/freely adjusted total sensitivity and conventional fundus autofluorescence. The mean ± SD difference in area between the 2 modalities was −0.479 ± 2.92 mm^2^ (*P* = .39). In a previous study for measuring areas of poorly demarcated questionably decreased autofluorescence, ICC for intergrader agreement was 0.715 (0.415, 0.875) using the semi-automated software tool (Heidelberg RegionFinder) and the ICC for intragrader agreement was 0.875 (0.715, 0.948) using the same semi-automated software tool (Heidelberg RegionFinder).^14^

### Qualitative Measures

Focus and clarity were determined as adequate by the graders in all 18 eyes for images obtained with freely adjusted total sensitivity and laser power of 25% and 100%, respectively. The image quality for 3 eyes was judged as fair for images acquired with 25% laser power/total sensitivity 87 (Supplemental Table 2; Supplemental Material available at AJO.com). Comparing the images obtained with the 3 different acquisition parameters, κ was 1.00 for the presence of flecks and for grading of a homogeneous or heterogeneous background signal, respectively. However, there were differences in grading increased autofluorescence at lesion edges between the images obtained with 25% laser power/total sensitivity 87, 25% laser power/free adjusted total sensitivity, and conventional fundus autofluorescence (Supplemental Table 2).

## Discussion

Several factors were considered during the design of the ProgStar study with regard to the use of fundus autofluorescence imaging.^10^ First, the research team weighed the risks of the elevated concentration of N-retinylidene-N-retinyethanolamine/lipofuscin found in Stargardt macular dystrophy, which is believed to be a contributor to cell death, and the possibility that the application of high-intensity short-wavelength blue light during fundus autofluorescence imaging could accentuate this toxicity.^11^, ^13^ This becomes particularly relevant in a natural history study, as serial imaging will be performed at regular intervals, and the imaging procedure itself may potentially contribute to N-retinylidene-N-retinyethanolamine accumulation and/or photooxidation.^13^ Second, although all ophthalmic imaging instruments are designed to operate well below safe retinal light damage thresholds, it must be noted that damage thresholds are derived from data using healthy retinas, whereas diseased retinas may have lower light damage thresholds.^13^, ^15^ Thus, there is a potential safety concern with the use of high laser powers.

Another potential benefit of the short-wavelength reduced-illuminance autofluorescence imaging approach is that it is a more comfortable imaging experience compared to conventional fundus autofluorescence imaging. The authors have anecdotal evidence of patients within the ProgStar study who questioned their continued participation owing to potential phototoxicity from fundus autofluorescence light exposure but were reassured by the reduced light intensities used in the short-wavelength reduced-illuminance autofluorescence imaging approach.

However, there was concern that by using less laser power or differences in sensitivity compared to conventional fundus autofluorescence, we would sacrifice image clarity and/or reduce accurate detection of all types of lesion and thereby compromise accurate measurement of progression, including assessment of total lesion area(s).

Owing to these aforementioned uncertainties, we conducted this study to directly compare 3 fundus autofluorescence imaging protocols (2 short-wavelength reduced-illuminance autofluorescence imaging parameters and conventional fundus autofluorescence) in a cohort of 18 patients with Stargardt macular dystrophy. In this cross-sectional study, we found no significant difference in the determination of the presence or absence of definitely decreased autofluorescence, or in area of definitely decreased autofluorescence, among the 3 autofluorescence image acquisition techniques. This finding is reassuring that reducing the illumination intensity will not compromise accurate detection and measurement of definitely decreased autofluorescence.

There was also good agreement in grading the presence and absence of poorly demarcated questionably decreased autofluorescence across all 3 image acquisition techniques; however, there was a difference in the area of poorly demarcated questionably decreased autofluorescence measured from images acquired by the different techniques. The largest mean area was observed using 25% laser power and 87 total sensitivity, which might be explained by the lower contrast that is typically observed in these images (Figure 1). However, such variability in grading lesion areas in poorly demarcated questionably decreased autofluorescence has also been observed in a previous pilot study published by Kuehlewein and associates, which evaluated the grading protocols for ProgStar^14^: fundus autofluorescence images obtained with 100% laser power and freely set total sensitivity (conventional fundus autofluorescence) were graded twice by different graders, and the main difficulty was the determination of borders in lesions with a black level close to the lowest threshold to be considered poorly demarcated questionably decreased autofluorescence. The ICC for intergrader agreement was 0.715 (0.415, 0.875) using the semi-automated software tool (Heidelberg RegionFinder) and the ICC for intragrader agreement was 0.875 (0.715, 0.948). In our study herein, the ICCs varied from 0.268 (0.000, 0.730), in the comparison between 25% laser power/freely adjusted total sensitivity and conventional fundus autofluorescence, to 0.821 (0.659, 0.983) when comparing 25% laser power/87 total sensitivity to conventional fundus autofluorescence. This finding seems counterintuitive, as we expected that the fixed setting of 25% laser power and total sensitivity of 87 would lead to lower ICCs in comparison with the other settings that allowed the photographer to adjust the total sensitivity. The increase in laser power appears to almost always necessitate a reduction in total sensitivity in order not to overexpose the resulting image, and therefore, images with higher laser power/lower total sensitivity are similar to those acquired with lower laser power/higher total sensitivity (Figure 1). We therefore believe that the variation and differences, both for ICCs and determined lesion areas in poorly demarcated questionably decreased autofluorescence cases, are due to the difficulty in delineating a lesion with poorly demarcated borders reliably and reproducibly, rather than in use of the short-wavelength reduced-illuminance autofluorescence imaging method per se.

Also, the concordance of qualitative measures such as the presence/absence of flecks and background heterogeneity was excellent, while differences in the grading of presence of hyperfluorescence at the lesion edge might be attributable to the differences in contrast.

The finding that short-wavelength reduced-illuminance autofluorescence imaging was comparable to conventional imaging for definitely decreased autofluorescence in our cross-sectional study may also have implications for its adoption in routine patient care. However, it will be important to also undertake a longitudinal study to determine how the imaging methods compare over time in assessing progression—especially in areas of more subtle patterns of altered FAF—in terms of its use in monitoring patients in clinic over time, but moreover for the purposes of clinical trial endpoint design.

Our study has some limitations. First, the number of eyes was small and thus, we could not assess differences between fundus autofluorescence acquisition methods for less common Stargardt fundus autofluorescence patterns, such as well-demarcated questionably decreased autofluorescence. Second, though the same photographer acquired the images with all 3 methods, we could not necessarily ensure that differences between the images were due to only the acquisition method, and not due to variability in the photographer's technique. Third, despite a break of at least 5 minutes between single image acquisitions, and also starting with the minimum laser power (25% laser power), there might have been a degree of bleaching effect that theoretically could affect the decreased autofluorescence categorization. It would be helpful if the grading software tool (Heidelberg RegionFinder) provided actual gray levels of the lesions to the graders.

A quantitative autofluorescence imaging and analysis method does exist^16^; however, bleaching of photoreceptors is a requirement for accurate analysis, and such an approach counteracts the underlying conceptual benefits of short-wavelength reduced-illuminance autofluorescence imaging.

Strengths of our study include the image acquisition by experienced, certified photographers at a single center using the same device, the fact that all image modalities for a given subject were obtained by the same photographer, and the application of standardized grading procedures by certified, experienced reading center graders.

In summary, short-wavelength reduced-illuminance autofluorescence imaging is a well-tolerated method to acquire fundus autofluorescence images, both clinically and, particularly, in the context of natural history studies where regular serial imaging is being undertaken. The acquisition of an image of good contrast by the photographer is of paramount importance for reliable grading results; our study provides valuable data that this can also be achieved by the use of short-wavelength reduced-illuminance autofluorescence imaging with freely adjusted total sensitivity. The ProgStar studies will shed further light into which types of decreased autofluorescence may serve as potential and reliable outcome measures. It may be speculated that in patients exhibiting lesions where fundus autofluorescence is relatively challenging from a measurement perspective (eg, poorly demarcated questionably decreased autofluorescence), other imaging modalities may be better suited to track disease progression, including spectral-domain optical coherence tomography.^17^, ^18^

## Figures and Tables

**Table 1:** Grading of Absolute Presence or Absence of 3 Different Lesion Categories of Decreased Autofluorescence Acquired With 3 Different Image Acquisition Settings

Acquisition Parameters	Number of Eyes With Definitely Decreased Autofluorescence
Laser power 25%, 87 total sensitivity	
Absent	3
Present	15
Laser power 25%, freely adjusted total sensitivity	
Absent	3
Present	15
Laser power 100%, freely adjusted total sensitivity	
Absent	4
Present	14
	Number of Eyes With Well-Demarcated Questionably Decreased Autofluorescence
Laser power 25%, 87 total sensitivity	
Absent	17
Present	1
Laser power 25%, freely adjusted total sensitivity	
Absent	17
Present	1
Laser power 100%, freely adjusted total sensitivity	
Absent	17
Present	1
	Number of Eyes With Poorly-Demarcated Questionably Decreased Autofluorescence
Laser power 25%, 87 total sensitivity	
Absent	6
Present	12
Laser power 25%, freely adjusted total sensitivity	
Absent	6
Present	12
Laser power 100%, freely adjusted total sensitivity	
Absent	6
Present	12

**Table 2:** Distribution of Grading and Kappa Values (95% Confidence Limits) for Grading of Presence or Absence of 3 Different Lesion Categories of Decreased Autofluorescence Acquired With 3 Different Image Acquisition Settings (Where Applicable)

Acquisition Parameters	Presence/Absence of Areas of Definitely Decreased Autofluorescence			
Laser power 25%, freely adjusted total sensitivity		Laser power 100%, freely adjusted total sensitivity	
Absent	Present	Absent	Present
Laser power 25%, 87 total sensitivity				
Absent	2	1	3	0
Present	1	14	1	14
	Kappa (95% confidence limits) = 0.60 (0.10–1.00)		Kappa (95% confidence limits) = 0.82 (0.49–1.00)	
Absent	Present
Laser power 25%, freely adjusted total sensitivity	-------------------			
Absent	3	0
Present	1	14
			Kappa (95% confidence limits) = 0.82 (0.49–1.00)	

Acquisition parameters	Presence/Absence of Areas of Well-Demarcated Questionably Decreased Autofluorescence			
Laser power 25%, freely adjusted total sensitivity		Laser power 100%, freely adjusted total sensitivity	
Absent	Present	Absent	Present
Laser power 25%, 87 total sensitivity				
Absent	17	0	17	0
Present	1	0	0	1
	Kappa n/a^a^		Kappa = 1.0	
Absent	Present
Laser power 25%, freely adjusted total sensitivity	-------------------			
Absent	16	1
Present	0	0
			Kappa n/a^a^	

Acquisition parameters	Presence/Absence of Areas of Poorly Demarcated Questionably Decreased Autofluorescence			
Laser power 25%, freely adjusted total sensitivity		Laser power 100%, freely adjusted total sensitivity	
Absent	Present	Absent	Present
Laser power 25%, 87 total sensitivity				
Absent	5	1	5	1
Present	1	11	1	11
	Kappa (95% confidence limits) = 0.75 (0.42–1.00)		Kappa (95% confidence limits) = 0.75 (0.42–1.00)	
Absent	Present
Laser power 25%, freely adjusted total sensitivity	-------------------			
Absent	6	0
Present	0	12
			Kappa = 1.0	

a Kappa was not calculated when row/column marginal was zero.

**Table 3:** Measures of Agreement in Areas of Definitely Decreased Autofluorescence Among Images Acquired With 3 Different Acquisition Parameters

Acquisition Parameters	Statistic	Acquisition Parameters	
25% Laser Power/Total Sensitivity Freely Adjusted	100% Laser Power/Total Sensitivity Freely Adjusted
25% laser power, total sensitivity 87	Difference in area of DDAF, mean(SD)	0.16 (0.51) mm^2^ (*P* = .20)^a^	0.036 (0.175) mm^2^, (*P* = .40)^a^
% Outside limits of agreement	2/18 (11.1%)	1/18 (5.5%)
Intraclass correlation coefficient (95% CI)	0.957 (0.915–0.999)	0.995 (0.991–1.00)
25% laser power, total sensitivity freely adjusted	Difference in area of DDAF, mean(SD)	__________________	−0.124 (0.473) mm^2^, (*P* = .28)^a^
% Outside limits of agreement	__________________	1/18 (5.5%)
Intraclass correlation coefficient (95% CI)	__________________	0.964 (0.929–0.999)

CI = confidence interval; DDAF = definitely decreased autofluorescence.

a Paired *t* test.

**Table 4:** Measures of Agreement in Areas of Poorly Demarcated Questionably Decreased Autofluorescence Among Images Acquired With 3 Different Acquisition Parameters

Acquisition Parameters	Statistic	Acquisition Parameters	
25% Laser Power/Total Sensitivity Freely Adjusted	100% Laser Power/Total Sensitivity Freely Adjusted
25% laser power, total sensitivity 87	Difference in area of PD-QDAF, mean(SD)	0.59 (1.74) mm^2^, (*P* = .18)^a^	0.11 (0.133) mm^2^, (*P* = .72)^a^
% Outside limits of agreement	4/18 (22.2%)	2/18 (11.1%)
Intraclass correlation coefficient (95% CI)	0.494 (0.117–0.870)	0.821 (0.659–0.983)
25% laser power, total sensitivity freely adjusted	Difference in area of PD-QDAF, mean(SD)	__________________	−0.479 (2.92) mm^2^, (*P* = .39)^a^
% Outside limits of agreement	__________________	2/18 (11.1%)
Intraclass correlation coefficient (95% CI)	__________________	0.268 (0.000–0.730)

CI = confidence interval; PD-QDAF = poorly-demarcated questionably decreased autofluorescence.

a Paired *t* test.

## Appendix

Rupert W. Strauss, MD, FEBO, is a clinical research fellow in the Departments of Inherited Retinal Disease and Genetics at Moorfields Eye Hospital and the UCL Institute of Ophthalmology, University College London (United Kingdom), respectively. He is also an honorary fellow at the Wilmer Eye Institute, Johns Hopkins University Baltimore (USA), where he has been working in the same field.
