# Supplementary figures and images for: Comparison of Short-Wavelength Reduced-Illuminance and Conventional Autofluorescence Imaging in Stargardt Macular Dystrophy
Source: Am J Ophthalmol. 2016 Aug;168:269–78. doi: 10.1016/j.ajo.2016.06.003 (PMC4977015; doi:10.1016/j.ajo.2016.06.003)

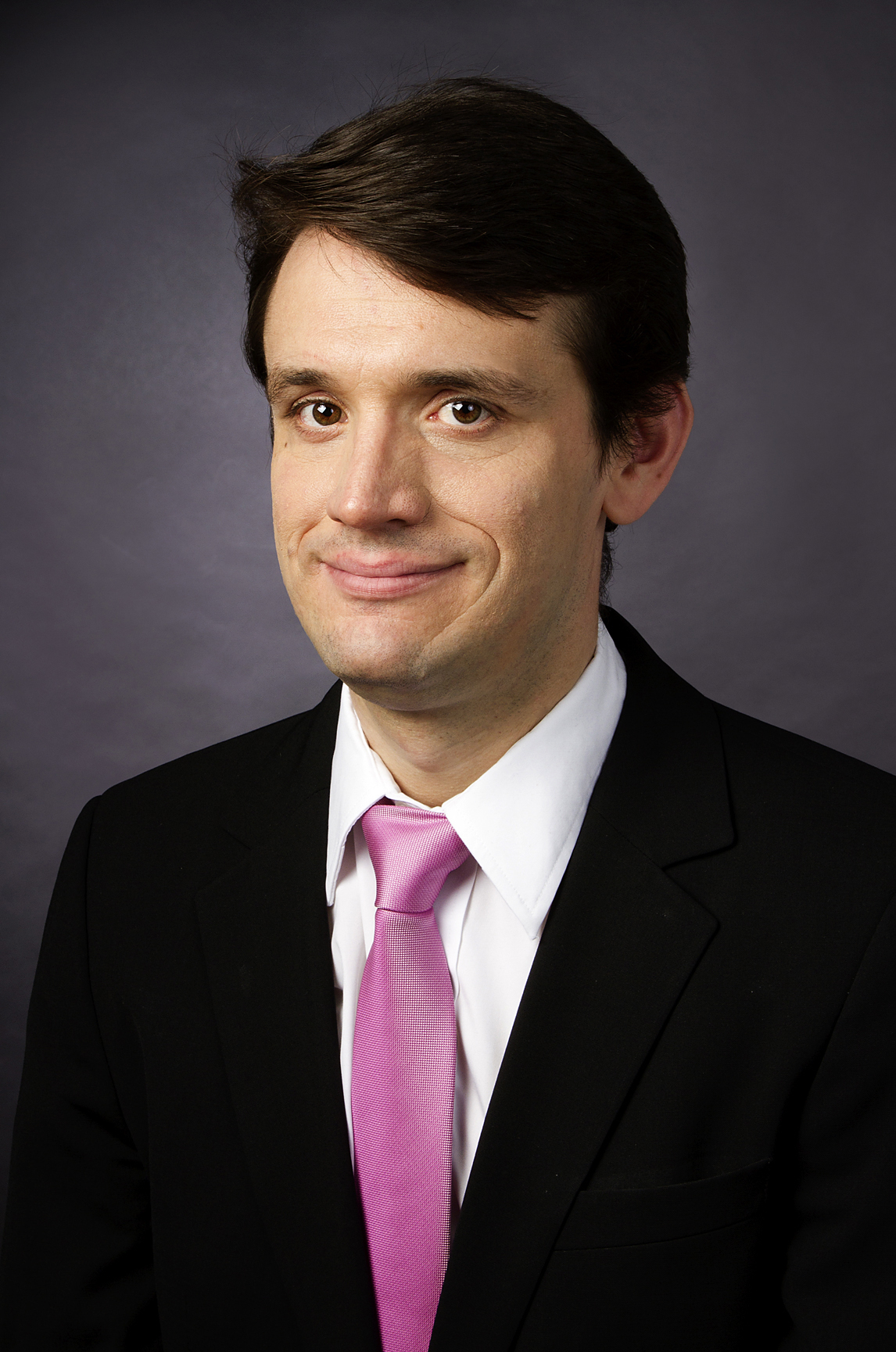

Supplement: Supplementary file 1 — Rupert W. Strauss, MD, FEBO, is a clinical research fellow in the Departments of Inherited Retinal Disease and Genetics at Moorfields Eye Hospital and the UCL Institute of Ophthalmology, University College London (United Kingdom), respectively. He is also an honorary fellow at the Wilmer Eye Institute, Johns Hopkins University Baltimore (USA), where he has been working in the same field. [file figs1.jpg]
