# Supplementary material for: Comparison of Short-Wavelength Reduced-Illuminance and Conventional Autofluorescence Imaging in Stargardt Macular Dystrophy
Source: Am J Ophthalmol. 2016 Aug;168:269–78. doi: 10.1016/j.ajo.2016.06.003 (PMC4977015; doi:10.1016/j.ajo.2016.06.003)
Supplement: Supplemental Table 1 [file mmc1.docx]

Supplemental Table 1. Demographic Characteristics of 18 Patients Enrolled in Study of Fundus Autofluorescence Acquisition Methods

| Characteristic |  |
| --- | --- |
| Number (%) of eyes | 18 |
| Right eyes | 7 (38.9%) |
| Left eyes | 11 (61.1%) |
| Age (y), mean (SD) | 38.3 (14.2) |
| Age of onset of symptoms (y) | 25.2 (15.6) |
| Sex, n (%) |  |
| Female | 11 (61.1%) |
| Male | 7 (38.9%) |
| Race, n (%) |  |
| White/Middle Eastern | 15 (83.3) |
| Black | 1 (5.6) |
| Asian/Indian | 2 (11.1) |
