# Supplementary material for: Comparison of Short-Wavelength Reduced-Illuminance and Conventional Autofluorescence Imaging in Stargardt Macular Dystrophy
Source: Am J Ophthalmol. 2016 Aug;168:269–78. doi: 10.1016/j.ajo.2016.06.003 (PMC4977015; doi:10.1016/j.ajo.2016.06.003)
Supplement: Supplemental Table 2 [file mmc2.docx]

Supplemental Table 2. Comparison of Focus and Clarity and Grading of Increased Autofluorescence at Lesion Edge Between the Images Obtained With 25% Laser Power/Total Sensitivity 87, 25% Laser Power/Freely Adjusted Sensitivity, and 100% Laser Power/Freely Adjusted Sensitivity

| Grading of Focus and Clarity in 3 Different Image Acquisition Parameters | | | | | | | | | | | | | | |  |  |  |
| --- | --- | --- | --- | --- | --- | --- | --- | --- | --- | --- | --- | --- | --- | --- | --- | --- | --- |
| Modality | | | 25% Laser Power, Freely Adjusted Total Sensitivity | | | | | | | | | | | |  |  |  |
| Laser power 25%, 87% sensitivity | | |  | | | | Adequate | | | | Fair | | | |  |  |  |
|  |  |  | Adequate | | | | 15 | | | | 0 | | | |  |  |  |
|  |  |  | Fair | | | | 3 | | | | 0 | | | |  |  |  |
| Modality | | | 100% Laser Power, Freely Adjusted Total Sensitivity | | | | | | | | | | | |  |  |  |
| Laser power 25%, 87% sensitivity | | |  | | | | Adequate | | | | Fair | | | |  |  |  |
|  |  |  | Adequate | | | | 15 | | | | 0 | | | |  |  |  |
|  |  |  | Fair | | | | 3 | | | | 0 | | | |  |  |  |
| Modality | | | 100% Laser Power, Freely Adjusted Total Sensitivity | | | | | | | | | | | |  |  |  |
| Laser power 25%, freely adjusted total sensitivity | | |  | | | | Adequate | | | | Fair | | | |  |  |  |
|  |  |  | Adequate | | | | 18 | | | | 0 | | | |  |  |  |
|  |  |  | Fair | | | | 0 | | | | 0 | | | |  |  |  |
| Grading of Presence of Increased Autofluorescence at Lesion Edge in 3 Different Image Acquisition Parameters | | | | | | | | | | | | | | | | | |
| Modality | | 25% Power, Freely Adjusted Total Sensitivity | | | | | | | | | | | | | | | |
| Laser power 25%, 87% sensitivity | |  | | | | Cannot grade | | | | No | | | | Yes | | | |
|  |  | Cannot grade | | | | 0 | | | | 1 | | | | 0 | | | |
|  |  | No | | | | 0 | | | | 9 | | | | 3 | | | |
|  |  | Yes | | | | 0 | | | | 2 | | | | 3 | | | |
| Modality | 100% Laser Power, Freely Adjusted Total Sensitivity | | | | | | | | | | | | | | | | |
| Laser power 25%, 87% sensitivity |  | | | Cannot grade | | | | No | | | | Questionable | | | | Yes | |
|  | Cannot grade | | | 0 | | | | 1 | | | | 0 | | | | 0 | |
|  | No | | | 0 | | | | 10 | | | | 2 | | | | 0 | |
|  | Yes | | | 0 | | | | 0 | | | | 0 | | | | 5 | |
| Modality | 100% Laser Power, Freely Adjusted Total Sensitivity | | | | | | | | | | | | | | | | |
| Laser power 25%, freely adjusted total sensitivity |  | | | | Cannot grade | | | | No | | | | Questionable | | | | Yes |
|  | Cannot grade | | | | 0 | | | | 0 | | | | 0 | | | | 0 |
|  | No | | | | 1 | | | | 8 | | | | 1 | | | | 2 |
|  | Yes | | | | 0 | | | | 2 | | | | 1 | | | | 3 |
